# Large animal model validation of a fenestrated balloon-expandable stent for treatment of aortoiliac occlusive disease

**DOI:** 10.1016/j.jvscit.2025.101950

**Published:** 2025-08-11

**Authors:** Sophia R. Pyeatte, Maxwell C. Braasch, John Cashin, Mohamed Zaghloul, Santiago Elizondo Benedetto, DeVaughn Rucker, Shahab Hafezi, Batool Arif, Guy M. Genin, Mohamed A. Zayed

**Affiliations:** a Division of Vascular Surgery, Department of Surgery, Section of Vascular Surgery, Washington University in St Louis, St Louis, MO; b Center of Cardiovascular Research Innovation in Surgery and Engineering, Washington University in St Louis, St Louis, MO; c Department of Biomedical Engineering, Washington University in St Louis McKelvey School of Engineering, St Louis, MO; d NSF Science and Technology Center for Engineering Mechanobiology, Washington University in St Louis McKelvey School of Engineering, St Louis, MO; e Division of Molecular Cell Biology, Washington University in St Louis School of Medicine, St Louis, MO; f Division of Surgical Sciences, Department of Surgery, Washington University in St Louis, St Louis, MO

**Keywords:** Aorto-iliac disease, Aortic device, Bench-top prototype, Large animal testing

## Abstract

The AortoIliac FENestrated (AIFEN) stent is a novel device that is designed for the endovascular management of aortoiliac occlusive disease while maintaining the natural aortoiliac anatomy and native bifurcation. Here, we report the first benchtop creation of this device through modification of a balloon-expandable covered stent and implanted in three large animal porcine aortoiliac segments. AIFEN stent placement was feasible and was maintained for 4 weeks in vivo. This first proof-of-concept AIFEN stent study demonstrates promising results and warrants further, rigorous evaluation to improve endovascular management of aortoiliac occlusive disease.

Peripheral artery disease is a significant health care burden.^1^^,^^2^ Endovascular treatment of chronic aortoiliac occlusive disease is limited to kissing iliac stents with 88% to 89.3% 1-year and 68.6% to 70% 5-year patency.^3^, ^4^, ^5^ Kissing iliac stents do not maintain native aortoiliac anatomy, which affects natural flow patterns in this region and impacts long-term patency.^6^^,^^7^

To address this area of persistent need, the concept of the AortoIliac FENestrated (AIFEN) stent was developed. A balloon-expandable covered stent is advanced and deployed into the aortoiliac segment from the distal abdominal aorta and into one of the common iliac arteries (CIAs). The fenestration in the device is oriented directly overlying the takeoff of the contralateral CIA.^6^ A separate balloon-expandable stent can then be advanced and deployed into the proximal contralateral CIA to achieve full luminal gain and to maintain the natural flow dynamics of the aortoiliac bifurcation anatomy.

Here, we demonstrate the feasibility of fabricating the AIFEN stent and percutaneously deploying it in a large animal aortoiliac anatomy. We also evaluate the preliminary efficacy of the stent to remain patent and provide sustained distal lower extremity perfusion.

## Methods

This study was approved by the Washington University in St Louis Institutional Animal Care and Use Committee, and animal care complied with the *Guide for the Care and Use of Laboratory Animals*.^8^ The AIFEN stent for management of the aortoiliac segment has received an issued United States patent.^9^ The AIFEN stents were fabricated by modifying LifeStream 10 mm × 37 mm balloon-expandable covered stents (BD). Following benchtop deployment to 10 mm, 7-mm fenestrations were created in the midshaft of the stents. These fenestrations were created using precise caliper measurement, and both the stent frame and covering were transected under magnification using scissors. The transected stent frame was kept covered along its edges. Radiopaque markers 2 mm in size were sutured to the edges of the fenestrations using 7-0 Prolene sutures to aid with fluoroscopic visualization of the fenestration during in vivo deployment. The AIFEN stents were recrimped using a three-dimensional-printed custom crimper and remounted on angioplasty balloons.

Implant feasibility was evaluated in vivo using three 60 to 70 kg adult male Yorkshire pigs. For all procedures, animals underwent general anesthesia, endotracheal intubation, and continuous hemodynamic monitoring. Pig 1 underwent percutaneous ultrasound-guided cannulation of the right femoral artery (CFA). The pig was then bolused with intravenous heparin (100 U/kg) to achieve an activated clotting time >250. Over a 0.035” Amplatz wire (Boston Scientific), a 12 Fr Brite Tip sheath (Cordis) was placed in the right CFA after serial dilations. A 5 Fr Omniflush catheter (AngioDynamics) was then advanced into the infrarenal abdominal aorta, and an aortogram and bilateral iliac angiograms were performed. A 10 mm × 37 mm balloon-expandable AIFEN stent was then advanced into the distal aorta via the right CIA with the midstent fenestration oriented directly adjacent to the origin of the left CIA. The stent was deployed, and poststenting balloon angioplasty was performed using a 10-mm Mustang balloon (Boston Scientific) to facilitate optimal stent opposition and conformability to the aortoiliac segment. Angiography was used to confirm appropriate alignment of the AIFEN fenestration relative to the left CIA.

In Pig 2, the same procedure was reproduced for AIFEN stent placement. Through a 9 Fr Brite Tip sheath (Cordis) in the left CFA, the fenestration in the AIFEN stent was catheterized retrograde from the left CFA using a 5 Fr angled glide catheter and 0.035” Glide wire (Terumo) under magnified fluoroscopy. The wire was then exchanged for an 0.035” Amplatz wire, and balloon angioplasty of the AIFEN fenestration was performed with a 7-mm Mustang balloon. A 7 mm × 25 mm LifeStream stent was advanced over the wire, into the proximal left CIA, and directly up to the AIFEN stent fenestration. The stent was positioned to minimize the gap up to the AIFEN, and also to ensure that the proximal end of the left CIA stent was not protruding into the fenestration. Poststenting balloon angioplasty was performed of the left CIA stent and AIFEN stent to optimize luminal expansion and radial fixation of the stents into the proximal iliac artery systems. Completion aortogram demonstrated brisk flow through the AIFEN and bilateral iliac arteries.

Finally, Pig 3 underwent the same procedure with implantation of an aorto-right iliac artery AIFEN stent and a left CIA stent. After completion angiography confirmed brisk flow in the AIFEN and bilateral iliac arteries, the sheaths were removed, and hemostasis was obtained using manual compression over the femoral cannulation sites. The pig survived and postoperatively received daily aspirin 325 mg and clopidogrel 75 mg. Weekly ultrasounds were performed to evaluate lower extremity inflow over a 4-week period. Although the iliac arteries could not be visualized due to overlying bowel gas, peak systolic velocities of the bilateral CFAs were captured and compared via Spearman’s correlation coefficient over time. For all pigs (1, 2, and 3) the aortoiliac arterial segments were harvested upon sacrifice and underwent gross inspection.

## Results

**Fig 1:**
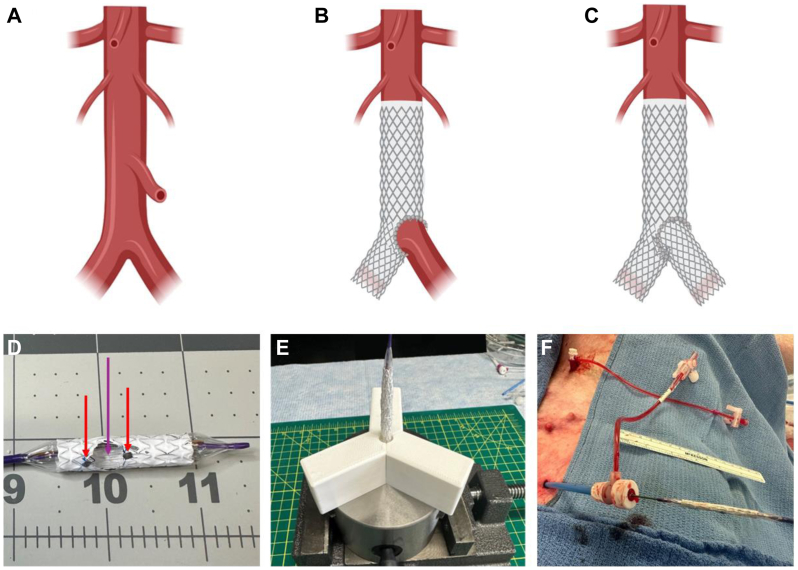
AortoIliac FENestrated (AIFEN) stent concept and prototype creation. **(A)** Aortoiliac anatomical segment; **(B)** AIFEN stent deployed in distal abdominal aorta and the proximal right common iliac artery with a fenestration oriented towards the proximal left common iliac artery (CIA) origin; **(C)** AIFEN and contralateral left CIA stents deployed; **(D)** AIFEN stent after benchtop fabrication with radiopaque clips (*red arrows*) and fenestration for the left CIA (*purple arrow*) marked; **(E)** AIFEN stent loaded in three-dimensional-printed crimper for crimping on factory-provided deployment balloon; **(F)** Crimped AIFEN stent prior to introduction into an animal model through percutaneous introducer.

Benchtop modification of LifeStream 10 mm × 37 mm balloon-expandable covered stents was successful in producing the AIFEN stent concept with a 7-mm fenestration and a radiopaque marker to highlight the edge of the fenestration (Fig 1).

**Fig 2:**
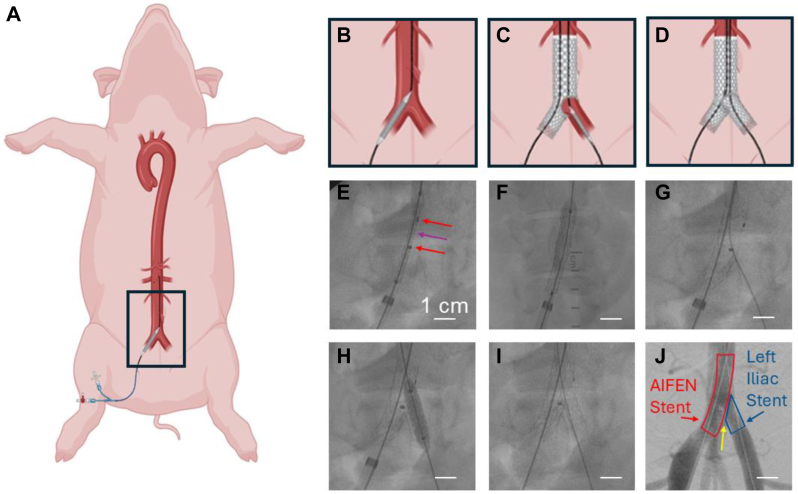
Placement of AortoIliac FENestrated (*AIFEN*) stent in a large-animal porcine aortoiliac segment. **(A)** Expanded view of porcine model with right common femoral artery (CFA) percutaneous access; **(B)** Diagram of introduction of AIFEN stent through 10 Fr percutaneous access in the right CFA; **(C)** Diagram of introduction of left common iliac artery (CIA) stent through 7 Fr percutaneous access of the left CFA; **(D)** Diagram of expansion of both the AIFEN and left iliac artery stent; **(E)** Intraoperative fluoroscopy demonstrating the undeployed AIFEN stent (*purple arrow*) and radiopaque markers around the fenestration (*red arrows*); **(F)** Intraoperative fluoroscopy demonstrating expansion of the AIFEN stent; **(G)** Intraoperative fluoroscopy demonstrating wire passed through the AIFEN stent fenestration; **(H)** Intraoperative fluoroscopy demonstrating expansion of the left iliac artery stent in the AIFEN stent fenestration; **(I)** Intraoperative fluoroscopy demonstrating both stents deployed; **(J)** Intraoperative fluoroscopy demonstrating completion angiogram demonstrating blood flow through both stents with *yellow arrow* indicating minimal gap but no overlap between the AIFEN and left iliac artery stents.

Pigs 1 and 2 both demonstrated the feasibility of percutaneous deployment of the AIFEN stent from a retrograde transfemoral approach. Additionally, in Pigs 2 and 3, the fenestrations were catheterized without technical difficulties from a retrograde contralateral femoral artery approach, which facilitated deployment of a contralateral CIA stent. In both pigs, the femoral access was uncomplicated, and the total procedure times were approximately 45 minutes long. Completion angiograms in both pigs demonstrated brisk flow in the bilateral iliac artery systems (Fig 2).

**Fig 3:**
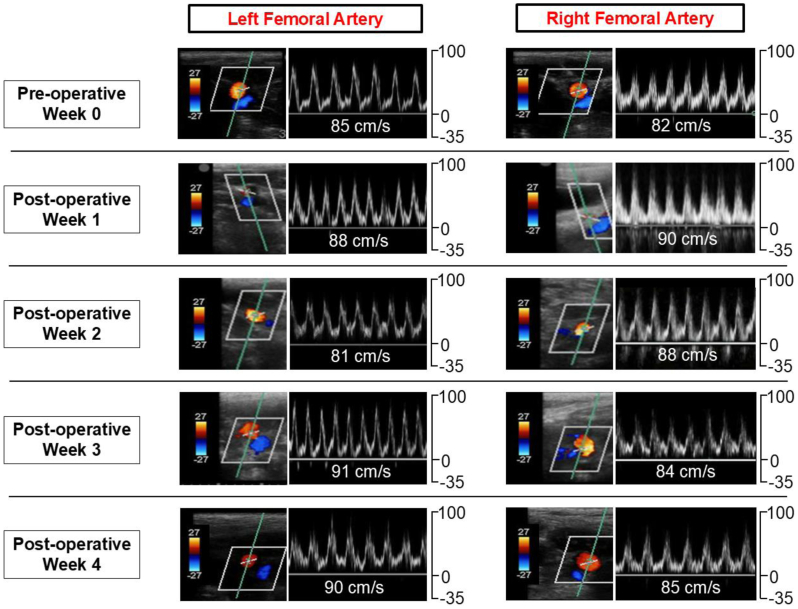
Arterial duplex monitoring of the bilateral femoral arteries pre- and post-AortoIliac FENestrated (AIFEN) stent placement. Arterial duplex ultrasonography with associated waveforms demonstrating peak systolic velocity from before AIFEN deployment in Pig 3 (Week 0) and weekly postoperatively until Week 4.

In Pig 3, all arterial ultrasound assessments demonstrated sustained CFA patency with no notable differences in laterality. Over the 4-week follow-up period, we observed no significant changes in the femoral artery inflow (Spearman’s correlation coefficient = −0.20) (Fig 3).

**Fig 4:**
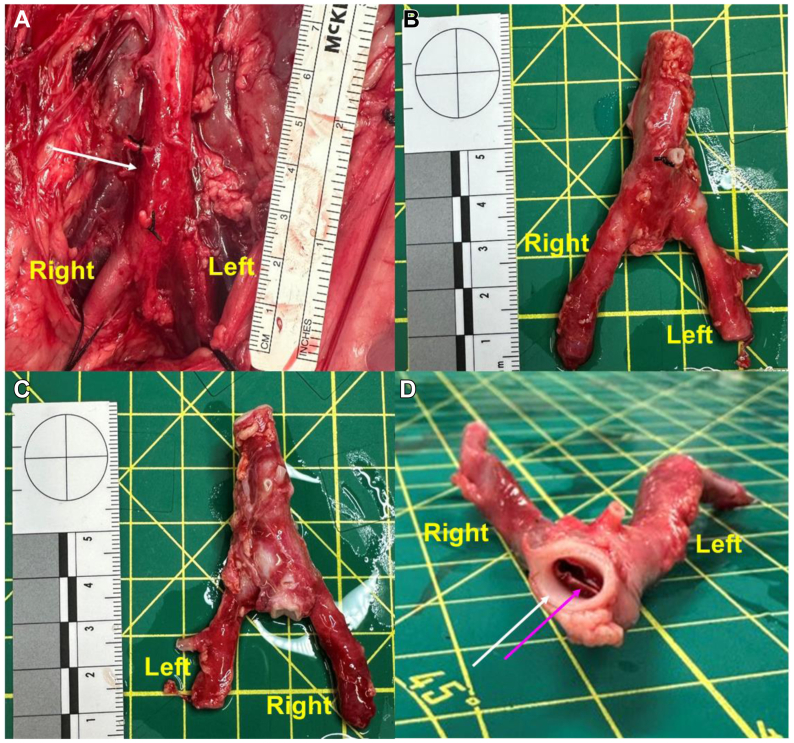
Gross examination of AortoIliac FENestrated (AIFEN) stent in vivo and ex vivo. **(A)** In situ aortoiliac segment with the AIFEN stent showing no gross trauma. *White arrow* indicating distal aorta; **(B** and **C)** Ventral and dorsal views of explanted aortoiliac segment with AIFEN stent demonstrating no gross arterial trauma; **(D)** Cranial view of the explanted aortoiliac system with the AIFEN stent through the aortic opening. *White arrow* indicates distal aorta, and *pink arrow* indicates proximal edge of the luminal AIFEN stent.

Gross examination of the aortoiliac systems and surrounding retroperitoneal tissue at the time of sacrifice demonstrated no obvious gross trauma to the aortoiliac segment. The luminal AIFEN stents were intact and properly positioned across the aortic bifurcations (Fig 4).

## Discussion

Here, we describe an innovation in the endovascular management of the aortoiliac segment. First, the AIFEN stent can be fabricated on a benchtop through modification of existing balloon-expandable covered stents and could safely be placed in the aortoiliac system of an adult large animal. Second, the AIFEN stent and contralateral left CIA stent in the AIFEN fenestration remained patent for 4 weeks with no deleterious impact on femoral arterial inflow.

Benchtop construction of the AIFEN stent was uncomplicated and reproducible. In theory, these stents could be made using predetermined luminal diameters that would be suitable for human aortoiliac anatomy. Both the AIFEN and contralateral left CIA stents were easy to position using standard fluoroscopic and angiographic techniques, and retrograde cannulation of the fenestration from the contralateral CFA was straightforward. The simplicity of the deployment of the AIFEN stent and the contralateral stent would facilitate ready adoption of the techniques.

Traditional kissing iliac stents placed in parallel “shotgun” configuration at the aortic bifurcation and into the bilateral common iliac arteries can artificially narrow the distal abdominal aorta prematurely and therefore disrupt arterial flow patterns.^6^^,^^7^ Additionally, the human aortoiliac bifurcation has a natural angle of 39° to 58° immediately after bifurcation.^10^ The AIFEN stent protects this natural angle of bifurcation.

There are some limitations to our study. First, improvements to the left CIA stent and the fenestration markings will occur as device manufacturing improves beyond these initial prototypes. Second, this pilot and feasibility study does not perform a comparative analysis to evaluate the efficacy of AIFEN compared with other stenting configurations. These future studies may provide additional insights about the potential advantages of the AIFEN stent. Third, this proof-of-concept study did not evaluate the potential risk of spillover lesions and intimal hyperplasia at the intersection of the AIFEN stent and the contralateral iliac artery stent. Future studies would be needed to rigorously analyze this potential facet of the AIFEN design. Lastly, this study was also not designed to evaluate long-term stent patency or risk of stent migration over time. As the AIFEN stent design is refined, these longer-term assessments will facilitate translation for human use.

## Conclusions

The AIFEN stent can successfully be constructed, implanted in a porcine model through percutaneous access without aortoiliac system injury, and remain patent in a 4-week follow-up period. The AIFEN stent demonstrates favorable results in a porcine model that warrants further investigation and rigorous analysis.

*The authors acknowledge the Washington University in St Louis Office of Technology Management for assistance with the relevant patent application filing and study seed funding.*

## Funding

This work was supported by the Center for Cardiovascular Research Innovation in Surgery and Engineering, 10.13039/100000002National Institutes of Health/10.13039/100000050National Heart, Lung, and Blood Institute T32HL170959, and seed funding from the 10.13039/100007268Washington University in St Louis Office of Technology Management.

## Disclosures

M.A.Z. and G.G. are co-founders of Caeli Vascular, Inc and Vascorra, LLC. Neither company is commercializing the endovascular technology reported in this manuscript. The remaining authors report no conflicts.
